# Steroid Cell Tumor of the Ovary in an Adolescent: A Rare Case Report

**DOI:** 10.1155/2013/527698

**Published:** 2013-02-21

**Authors:** Gokhan Boyraz, Ilker Selcuk, Zarife Yusifli, Alp Usubutun, Serdar Gunalp

**Affiliations:** ^1^Department of Obstetrics and Gynecology, Hacettepe University Faculty of Medicine, 06100 Ankara, Turkey; ^2^Department of Pathology, Hacettepe University Faculty of Medicine, 06100 Ankara, Turkey

## Abstract

Steroid cell tumors (SCTs) of the ovary are a rare subgroup of sex cord tumors, account for less than 0.1% of all ovarian tumors, and also will present at any age. These tumors can produce steroids, especially testosterone, and may give symptoms like hirsutism, hair loss, amenorrhea, or oligomenorrhea. For the evaluation of androgen excess, testosterone and dehydroepiandrosterone sulfate (DHEA-S) are the first laboratory tests to be measured. A pelvic ultrasound and a magnetic resonance imaging are useful radiologic imaging techniques. Although steroid cell tumors are generally benign, there is a risk of malignant transformation and clinical malignant formation. Surgery is the most important and hallmark treatment.

## 1. Introduction

Steroid cell tumors (SCTs) of the ovary are a rare subgroup of sex cord tumors, account for less than 0.1% of all ovarian tumors, and also will present at any age [1]. These tumors can produce steroids, especially testosterone, and may give symptoms like hirsutism, hair loss, amenorrhea, or oligomenorrhea [2]. These tumors have three subtypes, based on the cell of origin: stromal luteoma arising from ovarian stroma, Leydig cell tumor arising from Leydig cells in the hilus, and steroid cell tumor (not otherwise specified, or NOS) when the lineage of the tumor is unknown [3]. Here, we describe a patient of SCT, admitted to our clinic with symptoms of virilization.

## 2. Case Report

**Figure 1:**
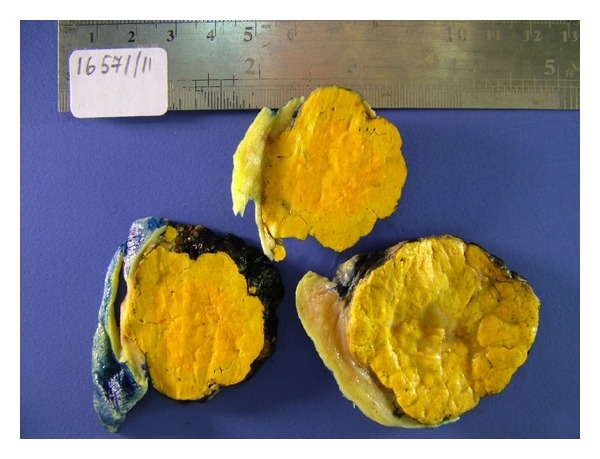
The tumoral mass has a yellow cut surface.

A 16-year-old virgin girl was admitted to our clinic with symptoms of acne, abnormal hair growth, and amenorrhea for 6 months. She had menarche at the age of 14, and since then, she was oligomenorrheic; her menses were between 40- and 45- day intervals. Although she had excess hair growth and acne formation previously, her symptoms had tremendously increased in that 6-month time period. Physical examination revealed a male pattern of hair growth in the face, anterior chest, legs, and arms; her breast development was normal, and she had no cliteromegaly. The sonography showed a 6 cm in diameter, cystic-solid, highly echogenic right ovarian mass, and there was no ascites. Her laboratory findings showed normal hemogram, electrolyte, creatinine, and liver enzyme levels. Her beta hCG level was negative, and Ca-125 and other tumor markers Ca19-9, Ca15-3, CEA, and alpha-fetoprotein (AFP) were within normal limits. We did not measure levels of AMH because of the young age of the patient. TSH, cortisol, prolactin, and DHEA-S (dehydroepiandrosterone sulfate) levels were normal, but testosterone and LDH (lactate dehydrogenase) levels were 94 ng/dL and 314 U/L, respectively; both of them were minimally elevated. Androstenedione, free testosterone, 17-OH progesterone, and sex hormone-binding globulin levels were also within normal limits. Estradiol level was not measured, and her growth rate was normal. Her endometrial thickness was 15 mm, and we did not perform an endometrial biopsy; the patient did not have much estrogenic symptoms, and she was virgin. The patient underwent an exploratory laparotomy, because of the possibility of malignancy; right ovarian cystectomy was performed, and frozen section revealed a steroid cell tumor. The other ovary was normal. The final pathology result showed a 6 cm steroid cell tumor without atypia and mitosis, and no necrosis was seen. Macroscopic examination showed a well-circumscribed tumoral mass measured as 6 × 4 × 3.3 cm. The neoplasm had a lobulated, solid, homogeneous, yellow cut surface (Figure 1).

**Figure 2:**
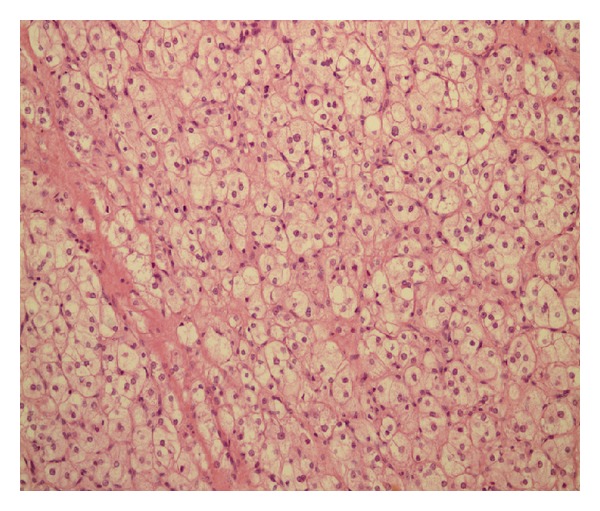
Cells with spongy cytoplasm focally separated by fibrous bands. No mitotic activity was seen.

In microscopic examination, cells were mostly arranged diffusely. But a minor fibromatous component and areas of hyalinization were also present. Necrosis, hemorrhage, and cystic degeneration were absent. No mitotic figure was observed (Figure 2).

**Figure 3:**
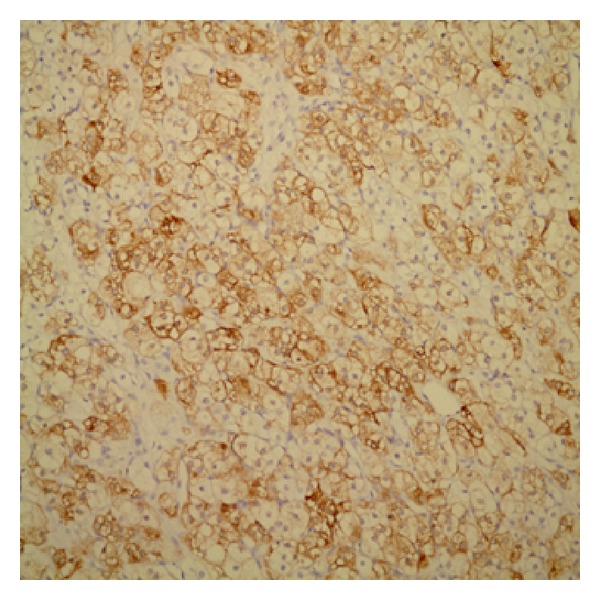
Diffuse membranous positivity with inhibin immunostaining.

**Figure 4:**
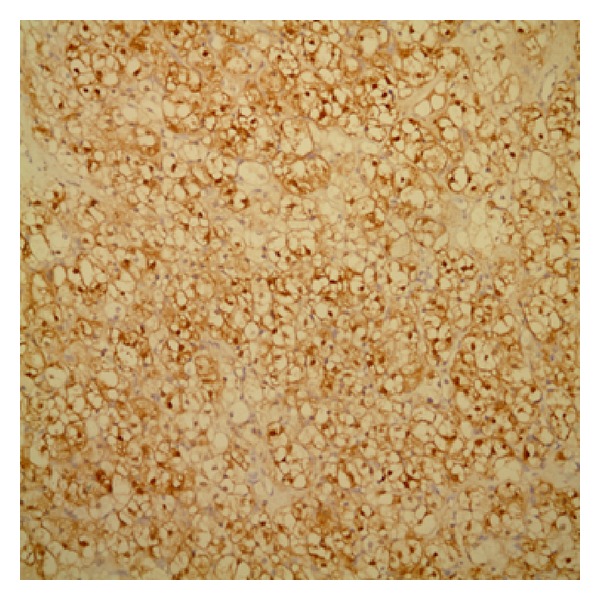
Diffuse cytoplasmic and nuclear positivity with calretinin immunostaining (×20).

By immunohistochemistry, neoplastic cells stained with inhibin and calretinin but negative with epithelial membrane antigen (EMA) (Figures 3 and 4).

Her testosterone level was 35.6 ng/dL at the postoperative 6th week. Her symptoms regressed at the postoperative 4th month, and her menses period came back. The patient is followed up closely and regularly with measurement of hormone levels and pelvic ultrasound as markers of recurrence.

## 3. Discussion

When a young woman comes with a rapid and sudden history of menstrual irregularity and virilization symptoms, an androgen excess situation especially suspicion for a tumor must come to mind immediately. Sex cord stromal tumors are developed from the sex cord and stromal components of the gonad [4]. Ovarian steroid cell tumors are grouped under sex cord tumors, and they are usually benign, unilateral, and formed by steroid cell proliferation. Between the three subtypes of SCTs, nearly 60% of the cases are steroid cell tumors [5]. For our patient, the tumor was also unilateral and benign (nonspecific inhibin and calretinin were positive without any mitosis). Steroid cell tumors show generally androgenic symptoms like amenorrhea, abnormal hair growth in the face and legs, and hair loss with a range of 12% to 50%, and that takes a long time to become evident [1]. Signs and symptoms of these tumors take place in an order: at the early stage, oligomenorrhea and minimal abnormal hair growth on body and, after that, amenorrhea, regression in the nature of breast, and other female external genitalia, hirsutism, acne, clitoral hypertrophy, and hair loss [6]. Although our patient was oligomenorrheic (40–45 days) and had excess hair growth, she showed significant and exaggerated symptoms for only a 6-month-period. 

For the evaluation of androgen excess, testosterone and DHEA-S are the first laboratory tests to be measured [7]. Elevation of testosterone levels above 200 ng/dL is the important diagnostic threshold level for the discrimination of androgen-secreting tumors and nonneoplastic lesions [8]. Our patient's testosterone level was 94 ng/dL at the initial testing, and that elevated level directed us towards the screening of pelvic structures. For elevated levels of testosterone, a pelvic ultrasound and a magnetic resonance imaging are useful radiologic imaging techniques for both ovary and adrenal glands [9]. They are usually unilateral, solid, slightly hyper or hypoechoic lesions as compared to the ovary and not associated with ascites [10]. We only performed a pelvic ultrasound because the DHEA-S level was normal and noticed a unilateral (right-sided), solid, hyperechoic ovarian tumor without ascites in abdomen as described in the literature.

Steroid cell tumors are generally unilateral and benign; malignancy is generally associated with identification of the histopathologic findings: two or more mitotic figures per 10 high-power fields 92% malignant, necrosis 86% malignant, size of 7 cm or larger 78% malignant, hemorrhage 77% malignant, and grade 2/3 nuclear atypia 64% malignant [11]. For our patient, the pathology result revealed a 6 cm size of unilateral mass, without atypia, necrosis, and mitosis.

Steroid cell tumors are generally composed of granular eosinophilic or vacuolated cytoplasm which is often positive for fat stains. In addition to these microscopic findings, steroid cell tumors would require immunohistochemical markers for the accurate diagnosis. Inhibin and calretinin are the most useful markers for the discrimination of sex cord stromal tumors from other tumors (Figures 3 and 4). And sex cord stromal tumors are mostly negative to EMA [12]. The histopathologic evaluation for our patient showed inhibin- and calretinin-positive and EMA-negative immunohistochemical findings, and that made the diagnosis easier.

Although steroid cell tumors are generally benign, there is a risk of malignant transformation and clinical malignant formation [6, 13]. Surgery is the most important and hallmark treatment, and complete excision of the tumor without chemotherapy or radiation which has no shown benefits could provide the regression of symptoms and disappearance of the virilizing effects [6, 14]. We performed laparotomy because of the risk of malignancy and the risk of rupturing the cyst while performing laparoscopy. We performed cystectomy instead of oophorectomy since the frozen section revealed no significant malignancy.

## 4. Conclusion

Steroid cell tumors are very rare, and androgenic symptoms with increased testosterone levels are important suspicious signs of a functional ovarian tumor. Malignancy is an important risk of steroid cell tumors, and pathologic evaluation is essential for the diagnosis of malignancy. Immunohistochemical testing is also helpful for the accurate diagnosis of a steroid cell tumor. Excision of the primary lesion is generally enough for the treatment of our patient. Our patient's symptoms becoming significant in a 6-month time, suddenly, in contrast to the literature, are the interesting part of our case, and the symptoms and signs of the patient were regressed in a 4-month period after the excision of lesion.
